# Risk Perception and Key Beliefs on Business Adaptation Behavior of Family Farmers: Empirical Evidence from Sichuan Province, China

**DOI:** 10.3390/bs15010086

**Published:** 2025-01-18

**Authors:** Yu Mou, Xiaofeng Li

**Affiliations:** Business School, Sichuan University, Chengdu 610064, China; my191001@163.com

**Keywords:** family farmers, adaptation behavior, risk perception, key beliefs, ordered logit

## Abstract

Family farmers face various business risks, including natural disasters, policies, technology adoption, and market uncertainties. This paper develops a conceptual framework for the formation mechanism of family farmers’ business adaptation behaviors by linking “risk perception, key beliefs, and adaptation behavior”. Using microdata from 363 family farmers in Sichuan Province, China, and applying ordered logit model regression analysis, we find that both risk perception and key beliefs positively influence family farmers’ business adaptation behavior. Key beliefs partially mediate this relationship, while risk preference negatively moderates the effect of risk perception on business adaptation behavior. Additionally, family farmers with higher farm profit levels and larger social networks are more likely to engage in business adaptation behavior. Family farmers who have higher education, are older, operate larger-scale farms, and are male exhibit stronger risk perceptions.

## 1. Introduction

A family farm in China refers to a new type of agricultural business entity that primarily relies on family members as the main labor force. It engages in scaled, intensive, and commercial agricultural production, with agricultural income serving as the primary source of household income. The terms “scaled”, “intensive”, and “commercial” highlight that family farms are larger in size compared to smallholder farms. In China, smallholder farms typically cover less than 10 mu, whereas crop-based family farms generally exceed 30 mu. Unlike smallholder farms, which are often self-sufficient, family farms focus more on efficient resource utilization to improve production efficiency, with most of their agricultural products intended for market sales (Du et al., 2020). As of March 2024, the number of family farms has grown to approximately four million in China. However, during the rapid growth of family farms, they also face various adverse business risks, such as extreme climate disasters, frequent outbreaks of pests and diseases, and fluctuations in agricultural product prices (Mou & Li, 2024). These risks can lead to a low output or revenue drops, undermining the economic viability of farms. Therefore, family farmers, driven by external threats, opportunities, or business strategies (Saebi et al., 2017), must consider adopting adaptive behaviors to cope with these risks. For instance, farmers may diversify their crops to manage market price fluctuations (Bowman & Zilberman, 2013), purchase insurance or join cooperatives to transfer risks (Zhang et al., 2019), and use different crop varieties to adapt to unfavorable weather conditions (Muraoka et al., 2021). Through effective adaptation behavior, they can reduce the impact of these risks and enhance the sustainable viability of their farms (Zhao, 2014; Song et al., 2019). Understanding the adaptive behaviors of farmers is beneficial for relevant departments to formulate corresponding policies for family farmers, which is of great significance in improving the economic output and sustainability of family farms. Psychology can help understand the response behavior process (Swim et al., 2011). Business adaptation behavior essentially involves the cognitive processes of individuals, including people’s subjective needs, values, belief systems, attitudes and perceptions, personalities, motivations, goals, and culture (Deressa et al., 2010; Nguyen et al., 2016; Norris, 2024; Wijethilake & Lama, 2018). Therefore, exploring the mechanisms behind family farmers’ business risk adaptation through behavioral science theories is of significant practical importance.

Existing research on farmers’ adaptive behaviors primarily focuses on how farmers respond to climate change or natural disasters, with particular emphasis on the influence of risk perception on adaptive behavior. Dang et al. (2014) emphasize that farmers’ climate change adaptation behavior is a psychological decision-making process; when farmers perceive potential threats, they tend to adopt various adaptive strategies. Risk perception is a crucial factor influencing farmers’ adaptive decisions, significantly impacting their behavior (Grothmann & Patt, 2005). The intensity of risk perception affects the choice of adaptive measures, with lower perceived risks and adaptive capacities reducing the likelihood of adopting adaptation practices (Below et al., 2012). For example, Lyu and Chen (2021), through a survey of farmers in the Shandong province of China, show that farmer’s risk perceptions of climate change and market risks—such as rainfall decreases, drought increases, and agricultural price drops—significantly influence their adaptive behaviors. Azadi et al. (2019) found that climate change beliefs, risk perception, psychological distance, and trust all affect farmers’ adaptation behaviors. Specifically, risk perception, trust, and psychological distance are key drivers of farmers’ adaptation behaviors. Zhu et al. (2023), based on the survey analysis of 414 large grain farmers in the Poyang Lake District of Jiangxi Province, also confirm that meteorological risk perception significantly affects information acquisition and adaptive behaviors.

However, according to the Theory of Planned Behavior (Ajzen, 1991; Fishbein & Ajzen, 2011), people’s adaptation behavior is not only influenced by risk perception but also by personal key beliefs. For example, de Leeuw et al. (2015), in a study on young people’s pro-environmental behavior, found that empathic concern indirectly influences behavioral intentions through its effects on behavioral, normative, and control beliefs. Similarly, Jacob et al. (2023), using structural equation modeling to analyze households’ adaptive behaviors in response to flooding disasters, show that behavioral, normative, and control beliefs are significant predictors of adaptive intentions, with normative beliefs being the most influential. In addition to risks associated with climate change and natural disasters, family farmers also face other business risks, including market (Lyu & Chen, 2021), policy (Mou & Li, 2024), and technology adoption risks (Muraoka et al., 2021). These factors significantly influence family farmers’ risk perceptions (Duong et al., 2019) and lead to different risk-adaptation behaviors. For instance, farmers may purchase agricultural insurance to mitigate policy, natural disaster, and market risks (Zhang et al., 2019); improve infrastructure, or introduce new technologies to reduce the impact of natural disasters (Schiavon et al., 2021; Yu & Wei, 2022); or select different crop varieties to cope with adverse weather conditions (Muraoka et al., 2021). We define family farmers’ risks in business adaptation behavior as the measures taken by family farmers to address various risks, including natural disasters, policy shifts, improper technology adoption, and market uncertainties. These measures may include diversification strategies, purchasing insurance, and strengthening infrastructure, among others. However, existing research has paid little attention to the comprehensive risk perceptions of family farmers and the relationship between their risk perceptions, key beliefs, and business adaptation behaviors. Incorporating these different dimensions into the study of adaptive behavior allows for a deeper understanding of farmers’ actions in the face of business risks.

In summary, existing research mainly focuses on studying farmers’ risk perceptions and adaptation behaviors related to climate change or natural disasters, with little exploration of how family farmers perceive other business risks—including policy, market, and technology adoption risks—and how these perceptions influence their business adaptation behaviors. Additionally, traditional research often neglects the role of key beliefs in driving behavior, making it difficult to fully understand a person’s behavior patterns and the mechanisms behind their formation. Therefore, this paper broadens the concept of risk perception to include various family farm business risks and constructs a framework to examine how risk perceptions and key beliefs influence family farmers’ adaptive behaviors.

## 2. Theoretical Hypothesis

### 2.1. Risk Perception on Business Adaptation Behavior of Family Farmers

Risk perception refers to an individual’s assessment of the probability of a particular event and its consequences or a subjective estimation of the nature of a threat and its severity (Sjöberg, 1998). In this study, family farmers’ risk perception is defined as their judgment of business risks and their perception of the severity of these risks on farm performance. By identifying and assessing business risks, family farmers can implement adaptation behaviors to minimize losses. Perception is a prerequisite for behavior, as Nguyen et al. (2016) emphasized that belief systems are foundational for farmers in assessing the degree and nature of external threats. Zhu et al. (2023) found that risk perception has a positive impact on the adaptation behavior of large grain farmers to meteorological disasters. In other words, the high level of risk perception of large grain growers can enhance the awareness of adaptation behavior, while a lower level of risk perception reduces this awareness. Grothmann and Patt (2005) examined the importance of psychological factors in adaptation decision-making in urban Germany and rural Zimbabwe and found that risk perception and perceived adaptive capacities were the most important bottlenecks. Lyu and Chen (2021) argued that if farmers perceive risks in a timely and accurate manner, it will lead to stronger adaptation intentions and more adaptive behaviors. In addition, the perception of market risks and farmers’ socio-economic characteristics also affect their adaptation actions. Therefore, the following hypothesis is proposed:

**H1:**  *Family farmers with higher risk perception are more likely to adopt business adaptation behaviors.*

### 2.2. The Mediating Effect of Key Beliefs

Personal key beliefs refer to the evaluation of a behavior’s positivity, the perceived social pressure to perform the behavior, and the perceived ease of performing the behavior (Armitage, 2001). Key beliefs can be categorized into three types: behavioral beliefs, normative beliefs, and control beliefs (Ajzen, 1991; Jacob et al., 2023). According to Ajzen’s definition (Ajzen, 1991), when applied to the key beliefs of family farmers, behavioral beliefs refer to the degree to which family farmers adopt business adaptation behaviors, including their views on the potential positive and negative outcomes of such behaviors. Normative beliefs refer to the influence of pressures from the government, society, family, and other factors on family farmers’ adoption of risk-adaptation behaviors. Control beliefs refer to their perceptions of the ease or feasibility of adopting adaptation behaviors in response to weather disasters. Family farmers who recognize and believe in the changing risks are more inclined to engage in adaptation behaviors and exhibit greater enthusiasm in implementing them. In other words, the stronger the key beliefs of farmers, the more likely they are to actively and proactively adopt adaptation behavior (Jacob et al., 2023; Borges et al., 2016). Moreover, in the study of the relationship between perception and beliefs, some research shows that perception precedes the formation of beliefs (Maftei & Holman, 2021; Beata Zarzycka et al., 2023); van Valkengoed et al. (2024) argued that risk perception positively influences efficacy beliefs, which, in turn, affect adaptation behaviors. When faced with various business risks, family farmers develop beliefs about adaptation behaviors through risk perception, motivating them to make decisions that align with these risks. In summary, key beliefs mediate the relationship between risk perception and the business adaptation behavior of family farmers. Therefore, the following hypothesis is proposed:

**H2:**  *Key beliefs mediate the relationship between risk perception and the adoption of business adaptation behavior by family farmers.*

### 2.3. The Moderating Effect of Risk Preference

Risk preference—also known as risk attitude, risk appetite, or risk propensity—is the amount and type of risk individuals are willing to take (Weber, 2010). In this study, risk preference is defined as the fundamental attitude that family farmers hold when facing risks in the family farm business processes. Regarding the relationship between risk preference, risk perception, and decision-making behavior in cognitive psychology, risk-averse individuals tend to be cautious in their behavioral decision-making, considering the results from the perspective of “avoiding disasters,” which leads them to make relatively conservative behavioral choices (Li & Huo, 2021). Meraner and Finger (2017) found that risk preference depends on the environment, meaning it varies across different decision-making areas at the farm level. Their analysis indicated that risk-averse farmers are more likely to prioritize risk management strategies within the farm rather than those outside the farm. Furthermore, Wan et al. (2023) found that risk preference influences individual decision-making, and the interaction between risk perception and risk preference impacts farmers’ farmland transfer-out behavior. Based on these findings, this study posits that risk preference has a moderating effect. Specifically, the risk preference of family farmers can indirectly influence their business adaptation behavior by affecting their risk perception. Moreover, a higher level of risk preference may decrease the likelihood of actively and proactively adopting business adaptation behaviors. In summary, the following hypothesis is proposed:

**H3:**  *Risk preference negatively moderates the relationship between risk perception and the adoption of business adaptation behavior by family farmers.*

**Figure 1:**
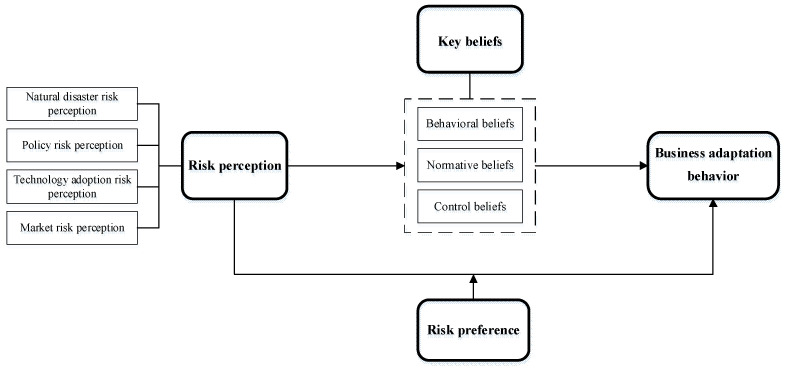
Conceptual framework.

Based on the above, the following theoretical model is proposed, as shown in Figure 1.

## 3. Method, Variables, and Data Sources

### 3.1. Method

This study employs an ordered logit model (Verbeek, 2008) to examine the impact of family farmers’ risk perception on their adoption of business adaptation behavior. Assume that the dependent variable of family farmers’ adaptation behavior, denoted as *Y*, is influenced by various factors. The effect of risk perception on farmers’ adaptation behavior is tested through the following regression model (1):

(1) $$Y=\alpha +\beta_{1}X+\lambda Contros+\varepsilon$$

where *Y* is the farmer’s adaptation behavior; *X* is the independent variable of risk perception; Contros is the control variable; α is the constant term, ε is the error term, $\beta_{1}$ is the estimated coefficient of the independent variable.

The mediating models (2) and (3) are as follows:

(2) $$M=\alpha +\beta_{2}X+\lambda Contros+\varepsilon$$

(3) $$Y=\alpha +\beta_{3}X+\beta_{4}M+\lambda Contros+\varepsilon$$

where M represents the mediating variable of key beliefs of family farmers, $\beta_{2}$, $\beta_{3}$, and $\beta_{4}$ are the coefficients to be estimated.

The moderating model (4) is as follows:

(4) $$Y=\alpha +\beta_{5}X+\tau_{1}\ln R+\tau_{2}X\times LnR+\lambda Contros+\varepsilon$$

where R represents the moderating variable of risk preference, X×LnR is the interaction term of risk perception and risk preference variables; if the coefficients $\beta_{5}$ and $\tau_{2}$ are significant, it suggests that risk preference impacts the effect of risk perception on farmers’ business adaptation behavior.

### 3.2. Variables

The variables and the measurement items in this study are presented in Table 1.

- Dependent variable: Business adaptation behavior. The selection of indicators for family farmers’ risk business adaptation behavior is based, on the one hand, on existing research (Lyu & Chen, 2021; Talanow et al., 2021) and, on the other hand, on the risk-adaptation behaviors that farmers consider most common and capable of addressing multiple risks in our survey reports. There are five types of risk-adaptation behaviors adopted by family farmers, namely, “adopting 0 kind”, “adopting 1 kind”, “adopting 2 kinds”, “adopting 3 kinds”, and “adopting 4 kinds”. The value for each of these behaviors is assigned one point, and the scoring interval is [0, 4].
- Independent variable: Risk perception. The scale measures risk perception across four dimensions: natural disaster risk perception, policy risk perception, technology adoption risk, and market risk perception based on the research (Meraner & Finger, 2017; Ullah et al., 2015). It assesses both the perceived severity and likelihood of these risks. The average score from the scale is used to calculate the risk perception for family farmers, and a higher average score indicates a higher level of risk perception among family farmers.
- Mediating and moderating variable: Key beliefs and Risk preference. Drawing from the key belief scales used in existing research on adaptation behavior (Jacob et al., 2023; Li & Shi, 2019). The key beliefs of family farmers are the average scores of behavioral belief, normative belief, and control belief scoring; the higher the average score, the stronger the key beliefs of the family farmer. The measurement of risk preferences refers to the independent self-report method (Iyer et al., 2019), measured by general risk preference, risk preference in the context of financial matters, and risk preference when it comes to the farmers’ farming management (Sulewski & Kłoczko-Gajewska, 2014). The average score of the scale represents the risk preference of family farmers. A higher score on the scale indicates a greater risk preference among family farmers.
- Control variable: The demographic characteristics, including gender, age, education, farm profit, farm size, availability of loans, and social network size, are selected as control variables (Lyu & Chen, 2021; Yin et al., 2020).

### 3.3. Data Sources

The research team conducted a survey of family farms in Sichuan Province, China, from March to June 2024. Using a disproportionate stratified sampling method, the survey encompassed crop-type and crop–livestock integrated family farms across five cities: Chengdu, Mianyang, Leshan, Neijiang, and Dazhou. These cities were selected to represent typical arable land terrains, including plains and hills. As shown in Table 2, we collected 402 questionnaires. After excluding those with missing critical indicators, 363 valid samples remained, resulting in a response rate of 90.3%. As shown in Table 3, the majority of family farmers in Sichuan Province are male (gender mean of 0.86). The average age of the family farmers is 46.2 years, with an average education level equivalent to junior high school (education level mean of 1.8). The average farm profit is 13.2 ten thousand yuan, and the average farm size is 78.3 mu.

## 4. Empirical Results

The article begins by examining the reliability and validity of the survey items related to risk perception, key beliefs, and risk preference. The Cronbach’s α values for these three scales are 0.805, 0.821, and 0.726, respectively, indicating reliable internal consistency of the questionnaire.

### 4.1. Correlation Analysis

We first calculated the Pearson correlation coefficients between the variables. As presented in Table 4, the results indicate a significant positive correlation between the risk perception and business adaptation behavior of family farmers (coefficient = 0.142, *p* < 0.05). Risk perception and key beliefs have a significant positive correlation (coefficient = 0.330, *p* < 0.01), as do key beliefs and business adaptation behavior (coefficient = 0.112, *p* < 0.05). These findings align with theoretical expectations and provide preliminary support for the related hypotheses.

### 4.2. Model Results

The variance inflation factors (VIFs) of the variables ranged from 1.010 to 1.631, all of which were below the critical threshold of 10, indicating that multicollinearity among the variables is not a concern. The parallelism test for the model regression revealed significance levels ranging from 0.774 to 0.856, all above 0.05, indicating that the ordered logit regression model is valid.

- Direct effect. As shown in Table 5, model one reports a coefficient value of 0.378 and significance below the 5% level, indicating that risk perception significantly positively influences the business adaptation behavior of family farmers, supporting Hypothesis H1. This suggests that a higher risk perception enables farm owners to proactively address internal and external risks and respond effectively to changes in natural, policy, technological, and market conditions, leading to more precise and effective adaptation strategies. Therefore, a higher level of risk perception by family farmers is associated with stronger business adaptation behaviors. Theoretically, family farmers who integrate resources, technology, and experience serve as a new type of agricultural business entity; their adaptation behaviors are informed by their risk assessments. The more risk perception, the more business adaptation behaviors are aimed at mitigating risks.
- Mediating effect. Models three and four present the regression analysis of the mediating variables. Model three indicates that key beliefs mediate the relationship between risk perception and business adaptation behavior, with risk perception significantly positively affecting key beliefs (coefficient = 0.140, *p* < 0.05). Model four shows that key beliefs positively influence business adaptation behavior (coefficient = 1.052, *p* < 0.01), supporting Hypothesis H2. Stronger key beliefs correlate with a higher adoption of business adaptation behaviors. Further analysis reveals that behavioral beliefs, normative beliefs, and control beliefs are all significant at the 1% level with positive coefficients, indicating that these beliefs significantly promote business adaptation behaviors. Positive attitudes towards agricultural adaptation and higher perceived controllability of these behaviors increase the likelihood of adoption. Additionally, adaptation behaviors are influenced by social groups such as family members, neighbors, village leaders, and government policy encouragement.
- Moderating effect. Model two shows that risk preference moderates the relationship between risk perception and business adaptation behavior. The interaction term between risk preference and risk perception has a significant negative effect on business adaptation behavior (coefficient = −0.602, significant at the 5% level). This indicates that while risk perception generally enhances business adaptation behavior, a higher risk preference diminishes the positive impact of risk perception. Farm owners with a higher risk preference may downplay or overlook risks, resulting in less adoption of adaptation behaviors. In other words, compared to a low-risk preference, the moderating effect of a high-risk preference leads to a more pronounced improvement in adaptation behavior due to risk perception. However, risk preference negatively moderates the effect of risk perception on adaptation behavior, thereby confirming Hypothesis H3.
- Control variables effect. Model four, shown in Table 5, demonstrates a high goodness-of-fit, indicating strong explanatory power. The analysis of control variables in model four reveals that farm profit significantly influences business adaptation behavior (coefficient = 0.150, significant at the 10% level). This suggests that increased capital enhances adaptation behavior. Regarding social capital, the frequency of interactions with others has a significant positive impact on business adaptation behavior (coefficient = 0.368, significant at the 1% level). This indicates that higher social capital, closer community ties, and more comprehensive agricultural information reduce future uncertainty.

In summary, risk perception directly influences the business adaptation behavior of family farmers and also affects it indirectly through key beliefs. Risk preference negatively moderates this relationship during the process of improving business adaptation, thereby supporting the proposed hypotheses in this study.

### 4.3. Robustness Tests

#### 4.3.1. Bootstrap Test

The Bootstrap method was used to test the significance of the mediating effect coefficient, addressing the limitations of stepwise regression, which is restricted to analyzing continuous dependent variables. It also avoids inconsistencies caused by different standard error formulas (Hayes, 2018). In this study, 5000 bootstrap samples were drawn from the original dataset, with a 95% confidence interval, to examine the mediating effect. As shown in Table 6, the confidence intervals of the direct and mediating effects have the same sign, indicating that key beliefs mediate the relationship between risk perception and the adoption of business adaptation behaviors by family farmers. This finding supports Hypothesis H2.

#### 4.3.2. Model Replacement

To ensure the robustness of the results, the regression analyses were re-run using both the OLS (Ordinary Least Squares) model and the Oprobit (Ordered Probit) model. As shown in Table 7, the results demonstrate that risk perception has a positive impact on the adoption of business adaptation behaviors by family farmers at the 1% significance level. This consistency across different models confirms the robustness of the regression results.

### 4.4. Heterogeneity Analysis

As shown in Table 8, male family farmers often exhibit stronger risk perceptions and core beliefs in agricultural production, which may be attributed to the more dominant roles they play in agricultural decision-making. They tend to be more attentive to external environmental changes, such as market fluctuations, weather risks, and policy shifts. In contrast, women may exhibit a relatively less pronounced risk perception and fewer beliefs, possibly due to family responsibilities or societal role expectations. Older family farmers generally possess more production and risk management experience, leading to a deeper understanding of agricultural risks. Having experienced more agricultural cycles and market fluctuations, they tend to possess stronger risk perception abilities. Family farmers with higher education levels are more likely to access modern agricultural information, such as weather forecasts, market analysis, and policy updates. They typically possess stronger analytical and decision-making abilities, leading to more significant risk perceptions and beliefs. Moreover, they are more likely to adopt new technologies, such as agricultural insurance and digital farming tools. Larger-scale family farmers typically have more resources, including capital, technology, and human support. Their operations are more aligned with modern agriculture, leading to a stronger perception of risk and beliefs. However, large-scale operations also face more complex potential risks, such as market fluctuations and supply chain disruptions, which may further heighten their sensitivity to risks.

## 5. Discussion and Policy Implications

This study provides comprehensive insights into the business adaptation behavior of family farmers, emphasizing the influence of risk perception, key beliefs, and risk preference. The findings align with and expand upon some prior research, offering a nuanced understanding of how these factors interact in the context of family farms and enriching the theoretical and practical implications.

The positive relationship between risk perception and business adaptation behavior highlights its critical role in motivating family farmers’ adaptive strategies, consistent with Zhu et al. (2023), Lyu and Chen (2021), and Meraner and Finger (2017), i.e., family farmers with heightened risk perception are more inclined to implement measures to mitigate internal and external threats. Moreover, the mediating analysis underscores the importance of key beliefs in translating risk perception into action. These beliefs significantly enhance farmers’ likelihood of adopting adaptive behaviors. Family farmers with stronger beliefs about the positive outcomes and feasibility of these actions exhibit greater enthusiasm for adaptation, consistent with Borges et al. (2016). In our study, we found that risk perception positively promotes family farmers’ key beliefs, which, in turn, affect adaptation behaviors. The moderating role of risk preference adds complexity to the relationship. While risk perception generally promotes adaptation behavior, higher levels of risk preference attenuate this effect. The heterogeneity analysis reveals notable variations in adaptation behavior based on demographic and operational characteristics. Male family farmers demonstrate stronger risk perception and beliefs, reflecting their dominant roles in agricultural decision-making and heightened awareness of external factors such as market volatility and policy shifts. Higher educational attainment equips farmers with advanced analytical and decision-making skills, enabling them to access and utilize modern agricultural tools like weather forecasts and digital technologies. Older farmers and larger-scale farm owners exhibit a deeper risk perception, which is consistent with Meraner and Finger (2017), likely due to their extensive experience with agricultural cycles and market dynamics and larger-scale farm owners, who are exposed to more complex risks and are more sensitive to these risks.

Based on the findings above, the following policy implications can be drawn: (1) Enhancing the information feedback to improve family farmers’ risk perception. Regular training should be conducted to equip farmers with knowledge about production-related risks and strategies for risk mitigation. Dynamic risk monitoring systems, coupled with the accurate and timely dissemination of production information, can improve farmers’ abilities to anticipate and respond to changing risks; (2) Strengthening key beliefs to promote risk adaptation. Governments and social organizations should leverage both online and offline communication channels to share success stories and the tangible benefits of adaptive measures. Highlighting positive outcomes will reinforce farmers’ behavioral and normative beliefs, and social networks and community platforms can also serve as effective tools for adaptation initiatives; (3) Promoting information symmetry and correcting perception biases. Information asymmetry and perception biases hinder effective adaptation. Governments should prioritize comprehensive risk detection and disclosure while providing farmers with tools and training to enhance their risk identification and management skills. Enabling farmers to make accurate judgments about business risks and the benefits of adaptation will reduce the influence of risk preferences on their decisions. Promoting information symmetry and correcting biases will encourage the adoption of more suitable and effective adaptation strategies; (4) Targeted interventions for diverse farmer groups. The heterogeneity analysis underscores the need for tailored interventions. For example, women and less-educated farmers may benefit from targeted training programs to strengthen their risk perception and beliefs. Similarly, risk-seeking farmers may require specific incentives or guidance to counteract their tendency to downplay risks.

## 6. Conclusions

This paper develops a conceptual framework for the formation mechanism of family farmers’ business adaptation behavior by linking “risk perception, key beliefs, and adaptation behavior”. The conceptual framework examines how risk perception and key beliefs influence the business adaptation behavior of family farmers. Compared to existing studies on risk perception related to climate change or natural disasters, we expand the concept of farmers’ risk perception to include a comprehensive definition encompassing natural disasters, policies, technology adoption, and market risks. Utilizing survey data from family farmers across five cities in Sichuan Province, we applied an ordered logit model to obtain regression results and conducted robustness tests and policy recommendations.

The study led to several key conclusions: First, risk perception positively influences the business adaptation behavior of family farmers; higher levels of risk perception increase the likelihood of adopting such behaviors. Second, key beliefs also positively impact business adaptation behavior, serving as a partial mediator in the relationship between risk perception and business adaptation behavior. Third, risk preference negatively moderates the effect of risk perception on business adaptation behavior. Additionally, at higher farm profit levels, both risk perception and key beliefs have a positive effect on the adoption of business adaptation behaviors, while a larger social network of relatives and friends strengthens the influence of risk perception on the adoption of these business adaptation behaviors. Heterogeneity analysis found that family farmers with higher education, older age, larger-scale farms, and who are male have a stronger risk perception.

Our study still has some limitations. In the future, we will consider sustainable business models and the perspectives of adaptive behavior processes, including risk aversion, tolerance, and acceptance. In addition, our research will also focus on classifying specific types of risks, examining how different risks interact with emotional factors and influence the adaptive behaviors of family farmers.

## Figures and Tables

**Table 1:** Variable measurement.

Variable	Measurement Item		Description
Dependent variable: Business adaptation behavior	Adopt new technologies		1 = adoption, 0 = no adoption
	Optimize farming time and use of pesticides or fertilizers		
	Adjust or diversify crop varieties		
	Purchase agricultural insurance		
Independent variable: Risk perception	Natural disaster	Performance losses caused by natural disasters	5-point scale: very low to very high
		Frequency of natural disasters	
	Policy	Performance losses caused by policy shifts	
		Likelihood of policy shifts	
	Technology adoption	Performance losses caused by inappropriate technology adoption	
		Likelihood of inappropriate technology adoption	
	Market	Performance losses caused by price fluctuations	
		Likelihood of price fluctuations	
Mediating variable: Key beliefs	Behavioral beliefs	Importance of adopting adaptation behaviors	5-point scale: very low to very high
		Benefits of promoting the adoption of adaptation behaviors	
	Normative beliefs	Extent to which policies guide the adoption of adaptation behaviors	
		Extent to which social factors guide the adoption of adaptation behaviors	
	Control beliefs	Feasibility of adopting adaptation behaviors	
		Perceived ease of use in adopting adaptation behaviors	
Moderating variable: Risk preference	General risk preference		5-point scale: very low to very high
	Risk preference in the context of financial matters		
	Risk preference when it comes to your farm and farming methods		
Control variables: demographic characteristics	Gender		1 = male, 0 = female
	Age		value (years)
	Education		0 = no school, 1 = primary school, 2 = junior high school, 3 = high school, 4 = junior college and above
	Farm profit		value (ten thousand yuan)
	Farm size		value (mu)
	Availability of loans		5-point scale: very difficult to very easy
	Social network size		5-point scale: very small to very large

Note: 1 mu = 1/15 hectare.

**Table 2:** The distribution of questionnaires.

City	Sample Size	Valid Samples	Sample Effective Rate (%)
Chengdu	158	142	89.9
Mianyang	67	61	91.0
Leshan	59	55	93.2
Nei jiang	62	56	90.3
Dazhou	56	49	87.5
Total	402	363	90.3

**Table 3:** Descriptive statistics.

Variable	Min	Max	Mean	Standard Deviation
Business adaptation behavior Y	0.0	4.0	2.1	0.9
Risk perception X	1.1	5.0	3.8	0.7
Key beliefs M	1.1	5.0	3.5	0.9
Risk preference R	0.0	5.0	2.5	1.5
Gender	0.0	1.0	0.86	0.18
Age	18.0	65.0	46.2	9.1
Education	0.0	4.0	1.8	0.5
Farm profit	3.5	40.0	13.2	6.4
Farm size	30.0	360.0	78.3	18.6
Availability of loans	1.0	5.0	2.7	1.3
Social network size	1.0	5.0	3.0	1.1

**Table 4:** Pearson correlation results.

Variable	Business Adaptation Behavior	Risk Perception	Key Beliefs	Risk Preference
Business adaptation behavior	1			
Risk perception	0.142 **	1		
Key beliefs	0.330 ***	0.112 **	1	
Risk preference	−0.202 ***	0.010	0.050	1

Note: *** *p* < 0.01, ** *p* < 0.05.

**Table 5:** Model regression results.

Variable	Model 1 (Y)	Model 2 (Y)	Model 3 (M)	Model 4 (Y)
Core explanatory variable: Risk perception X	0.378 ** (0.142)	0.927 *** (0.341)	0.140 ** (0.064)	1.052 *** (0.348)
Mediating variable: Key beliefs M				0.787 *** (0.127)
Moderating variable: Risk preference lnR		0.837 (1.039)		1.246 (1.055)
Risk perception X × Risk preference lnR		−0.452 * (0.271)		−0.602 ** (0.277)
Control variable				
Gender	0.375 (0.285)	0.386 (0.289)	−0.069 (0.130)	0.366 (0.295)
Age	0.083 (0.098)	0.086 (0.099)	0.060 * (0.025)	0.059 (0.101)
Education	−0.035 (0.086)	−0.042 (0.086)	−0.041 (0.039)	−0.010 (0.088)
Farm profit	0.149 * (0.086)	0.148 * (0.087)	0.015 (0.039)	0.150 * (0.088)
Farm size	0.156 (0.108)	0.155 (0.109)	0.037 (0.049)	0.123 (0.111)
Availability of loans	−0.052 (0.081)	−0.044 (0.081)	−0.032 (0.037)	−0.023 (0.083)
Social network size	0.327 *** (0.111)	0.361 *** (0.112)	−0.049 (0.050)	0.368 *** (0.114)
Pseudo R-Square/R-Square	0.091	0.146	0.030	0.224

Note: *** *p* < 0.01, ** *p* < 0.05, * *p* < 0.1.

**Table 6:** Mediating effect test (95% confidence interval).

Effect	Effect Value	SE	LLCI	ULCI
Direct effect	0.122 **	0.052	0.021	0.224
Indirect effect	0.036 **	0.017	0.004	0.071

Note: ** *p* < 0.05.

**Table 7:** Replacement model results.

Variable	OLS	Oprobit
Risk perception	0.323 ** (0.117)	0.564 ** (0.195)
Key beliefs	0.271 *** (0.041)	0.451 *** (0.071)
Risk preference	0.343 (0.0363)	0.642 (0.596)
Risk perception × risk preference	−0.178 * (0.094)	−0.316 ** (0.156)
Control variable	Yes	Yes
Pseudo R-Square/R-Square	0.225	0.113

Note: *** *p* < 0.01, ** *p* < 0.05, * *p* < 0.1.

**Table 8:** Grouped regression results.

Variable	(1)	(2)	(3)	(4)	(5)	(6)	(7)	(8)
	Female	Male	Age (l)	Age (h)	Education (l)	Education (h)	Farm Size (l)	Farm Size (h)
X	0.492	0.296 **	0.259 *	0.364 **	0.182	0.390 **	0.221 *	0.374 **
	(1.083)	(1.951)	(1.318)	(1.660)	(0.706)	(2.248)	(1.133)	(1.774)
M	0.319	0.634 ***	0.364 ***	0.939 ***	0.209	0.717 ***	0.502 ***	0.636 ***
	(0.894)	(4.819)	(2.215)	(4.876)	(0.947)	(4.825)	(2.995)	(3.463)
Control variable	Yes	Yes	Yes	Yes	Yes	Yes	Yes	Yes

Note: l represents below-average group, h represents above-average group. *** *p* < 0.01, ** *p* < 0.05, * *p* < 0.1.

## Data Availability

The data that support the findings of this study are available on request from the corresponding authors. The data are not publicly available due to privacy or ethical restrictions.
